# Risk factors for bone loss in patients with rheumatoid arthritis treated with biologic disease-modifying anti-rheumatic drugs

**DOI:** 10.1186/s13104-017-3086-7

**Published:** 2017-12-21

**Authors:** Hiroki Tawaratsumida, Takao Setoguchi, Yoshiya Arishima, Hideo Ohtsubo, Masaki Akimoto, Yasuhiro Ishidou, Satoshi Nagano, Eiji Taketomi, Nobuhiko Sunahara, Setsuro Komiya

**Affiliations:** 1 Department of Orthopaedic Surgery, Graduate School of Medical and Dental Sciences, Kagoshima University, Kagoshima, Japan; 2 The Near-Future Locomotor Organ Medicine Creation Course (Kusunoki Kai), Graduate School of Medical and Dental Sciences, Kagoshima University, 8-35-1 Sakuragaoka, Kagoshima, 890-8520 Japan; 3 Center for Rheumatic Diseases, Japanese Red Cross Kagoshima Hospital, Kagoshima, Japan; 4 Department of Hematology and Immunology, Graduate School of Medical and Dental Sciences, Kagoshima University, Kagoshima, Japan; 5 Department of Medical Joint Materials, Graduate School of Medical and Dental Sciences, Kagoshima University, Kagoshima, Japan; 6 Department of Orthopaedic Surgery, Japanese Red Cross Kagoshima Hospital, Kagoshima, Japan

**Keywords:** Biological disease-modifying anti-rheumatic drugs (bDMARDs), Osteoporosis, Rheumatoid arthritis (RA), Risk factors

## Abstract

**Objective:**

Osteoporosis is a complication of rheumatoid arthritis. We examined the risk factors for bone loss in rheumatoid arthritis patients receiving biological disease-modifying anti-rheumatic drugs. Lumbar spine and femoral neck bone mineral density was measured at two time points in 153 patients with rheumatoid arthritis managed with biological disease-modifying anti-rheumatic drugs. We examined patients’ variables to identify risk factors for least significant reduction of bone mineral density.

**Results:**

Least significant reduction of lumbar spine bone mineral density (≤ − 2.4%) was seen in 13.1% of patients. Least significant reduction of femoral neck bone mineral density (≤ − 1.9%) was seen in 34.0% of patients. Multiple logistic regression analysis showed that a risk factor for least significant reduction of the lumbar spine was high-dose methylprednisolone use. Multiple regression analysis showed that a risk factor for least significant reduction of the femoral neck was short disease duration. Our findings showed that a risk factor for femoral neck bone mineral density reduction was a short disease duration. These findings suggest that rheumatoid arthritis patients receiving treatment with biological disease-modifying anti-rheumatic drugs may benefit from earlier osteoporosis treatments to prevent femoral neck bone loss.

**Electronic supplementary material:**

The online version of this article (10.1186/s13104-017-3086-7) contains supplementary material, which is available to authorized users.

## Introduction

Rheumatoid arthritis (RA) is an autoimmune disease that promotes joint inflammation and destruction. It has been reported that 15–20% of RA patients are affected by osteoporosis of the hip and spine [1, 2]. Inflammation plays a key role in RA activity, as well as in bone resorption and osteoporosis [3]. The treatment of RA has advanced significantly by targeting key molecules including tumor necrosis factor alpha (TNF-α) or interleukin 6 (IL-6) using a group of drugs called biological disease-modifying anti-rheumatic drug (bDMARDs) [4–6]. There is also evidence suggesting that bDMARDs may have beneficial effects on bone metabolism and bone remodeling [3, 5, 7–13]. Transgenic mice expressing soluble TNF-α receptor were protected from bone loss caused by estrogen-deficiency-related osteoporosis [14]. The blockade of inflammatory cytokines in RA may therefore not only reduce inflammation but also generalized bone loss. In this study, we examined changes in the bone mineral density (BMD) of patients with RA being treated with bDMARDs. We aimed to identify specific patient factors associated with least significant reduction (LSR) of BMD in the lumbar spine and femoral neck.

## Main text

### Methods

#### Patients

We retrospectively examined the records of 153 consecutive patients with RA diagnosed using American College of Rheumatology/European League Against Rheumatism classification criteria [15]. All consecutive patients who underwent DXA scanning of the lumbar spine and femoral neck were included. Inclusion criteria for the study were that the patients (1) had undergone bDMARDs therapy for ≥ 1 year; (2) had undergone dual-energy X-ray absorptiometry (DXA) as a baseline study and again after 1 year and/or during long-term follow-up; and (3) had all parameters recorded in an electronic medical record. The patients for whom some of these data were missing were excluded from the study. All patients diagnosed and started medication at the Japanese Red Cross Kagoshima Hospital. When conventional DMARDs treatment led to a Disease Activity Score 28-C-reactive protein (DAS28-CRP) value of < 2.6 or a Simplified Disease Activity Index of ≤ 3.3, bDMARDs were not needed [16, 17]. If the disease activity was not lowered sufficiently to meet these criteria, therapy with bDMARDs was started. The choice of bDMARDs was made by the attending physician. The bDMARDs used for treatment in this study included infliximab, adalimumab, golimumab, etanercept, tocilizumab, and abatacept. All treatments were undertaken at the Japanese Red Cross Kagoshima Hospital, as previously reported [18]. Demographic and disease-related data were collected retrospectively from the medical records. We examined age, sex, disease duration, body mass index (BMI), average prescribed dose of methylprednisolone, serum C-reactive protein (CRP), Disease Activity Score 28-CRP, simplified disease activity index, duration of bDMARDs therapy, and the type of anti-osteoporosis drugs to identify risk factors for LSR of BMD. We calculated changes in BMD over a 12-month period. Anti-osteoporosis drugs were used according to the guideline for treating osteoporosis and glucocorticoid-induced osteoporosis [19–22]. In addition, if blood tests showed a significant reduction in the 1.25(OH)_2_ vitamin D level, a vitamin D formulation was administered. All patients gave written informed consent for their records to be published in this study.

#### BMD of the lumbar spine and femoral neck

BMD was examined between December 2011 and December 2013 by the Discovery DXA system (Hologic, Waltham, MA, USA). The BMD of the lumbar spine (L2–L4) and femoral neck (g/cm^2^) were measured. The mean duration between each BMD examination was 12 (range 11–13) months.

#### Criteria for reduction of BMD

Judgment of treatment results using BMD was offered by the Scientific Task Force Group of the International Osteoporosis Foundation (IOF) [23], which was based on the notion of least significant change (LSC). Therefore, the LSC is calculated by the coefficient of variation of BMD measurement, a value that exceeds the LSC can be recognized as a significant reduction in BMD during short duration. The LSR of BMD was used as the criterion for judging BMD reduction. Reduced BMD was defined as previously reported [23, 24]. The BMD coefficient of variation was 2.0% for the lumbar spine and 1.6% for the femoral neck [24]. The LSC with a one-tailed test would be 1.19 times for evaluation of individual cases, with 80% confidence. The LSR of BMD was ≤ − 2.4% for the lumbar spine and ≤ − 1.9% for the femoral neck [24].

#### Statistical analysis

Univariate and multivariate stepwise binomial logistic regression were performed to correlate demographic and patient-reported outcome metrics with changes in LSR of BMD. Because of the relatively small number of patients and the large number of confounding factors, we applied a stepwise variable selection method to identify significant factors, as previously described [25]. Stepwise variable selection is a method of fitting regression models in which choice of variables is performed by an automatic procedure. Because of potential confounding factors between the variables, we did not exclude any variables according to the results of the univariate analyses. In the stepwise model, all variables are included to the model at first but may also be removed until the current model is identical to the model estimated in the previous model. For a variable to enter or stay in the model, we defined a p value of 0.2 as cut-off for variables to enter in the model. p < 0.05 was determined as statistically significant. Analysis was performed using BellCurve for Excel (Social Survey Research Information Co., Ltd., Tokyo, Japan).

### Results

The demographic and clinical characteristics of the 153 patients who underwent DXA scanning of the lumbar spine and femoral neck are shown in Table 1. LSR of lumbar spine (≤ − 2.4%) BMD was seen in 13.1% (20/153) of patients, and LSR of femoral neck BMD (≤ − 1.9%) was seen in 34.0% (52/153) of patients. Scatter plots were shown in Additional file 1: Figure S1. Univariate analysis detected no risk factors for 12-month LSR of lumbar spine and femoral neck BMD (Table 2). Multiple stepwise binomial logistic regression analysis showed that a risk factor for bone loss of the lumbar spine was the use of high dose of methylprednisolone (Table 3). Multiple regression analysis showed that a risk factor for LSR of the femoral neck was a short disease duration (Table 3).

**Table 1:** Patient demographic and clinical characteristics

Age (year)	60.8 ± 10.5
Proportion of female	83.0%
Disease duration (year)	10.0 (5.0–17.0)
BMI	22.8 ± 3.4
Average dose of prednisolone (mg)	0.0 (0.0–2.0)
CRP (mg/dL)	0.12 (0.03–0.38)
DAS28-CRP	2.39 (1.62–3.47)
SDAI	6.07 (2.35–14.32)
MHAQ	4.0 (0.0–10.0)
Duration of bDMARDs therapy	4.0 (3.0–6.0)
Type of anti-osteoporosis drugs	PTH: 3.5% Bisphosphonate: 47.1% Others: 14.4% None: 35.3%
Change of lumbar spine BMD/12 months (g/cm^2^)	0.004 (− 0.080 to 0.018)
Percent of least significant reduction of lumber spine BMD	13.1% (20/153)
Change of femoral neck BMD/12 months (g/cm^2^)	− 0.001 (− 0.025 to 0.019)
Percent of least significant reduction of femoral neck BMD	34.0% (52/153)

*BMI* body mass index, *CRP* serum C-reactive protein concentration, *DAS28-CRP* Disease Activity Score-28-CRP, *SDAI* simplified disease activity index, *MHAQ* modified Health assessment questionnaire, *bDMARDs* biologic disease-modifying anti-rheumatic drugs, *PTH* parathyroid hormone, *BMD* bone mineral density

**Table 2:** Univariate analysis of risk factor for LSR of BMD

	Lumber spine		Femoral neck	
	Odds ratio	p value	Odds ratio	p value
Age (year)	0.98 (0.94–1.02)	0.28	0.98 (0.95–1.02)	0.34
Sex	0.71 (0.21–2.34)	0.57	2.25 (0.79–6.42)	0.13
Disease duration	1.03 (0.97–1.08)	0.34	0.96 (0.92–1.01)	0.10
BMI	1.04 (0.90–1.19)	0.61	0.97 (0.88–1.07)	0.53
Average dose of prednisolone	1.18 (0.99–1.41)	0.06	1.10 (0.95–1.27)	0.22
CRP	1.51 (0.99–2.30)	0.05	1.04 (0.70–1.53)	0.85
DAS28-CRP	1.33 (0.91–1.96)	0.14	1.00 (0.76–1.33)	0.99
SDAI	1.03 (0.98–1.08)	0.20	1.00 (0.97–1.04)	0.92
MHAQ	1.03 (0.95–1.11)	0.46	0.99 (0.93–1.05)	0.68
Duration of bDMARDs therapy	0.98 (0.81–1.19)	0.83	0.91 (0.79–1.05)	0.18
Type of anti-osteoporosis drugs	1.26 (0.77–2.05)	0.36	0.93 (0.66–1.32)	0.68

Univariate analysis detected no risk factors for 12-month LSR of lumbar spine (≤ − 2.4%) and femoral neck (≤ − 1.9%) BMD

*LSR* least significant reduction, *BMD* bone mineral density, *BMI* body mass index, *CRP* serum C-reactive protein concentration, *DAS28-CRP* Disease Activity Score-28-CRP, *SDAI* simplified disease activity index, *MHAQ* modified health assessment questionnaire, *bDMARDs* biologic disease-modifying anti-rheumatic drugs

**Table 3:** Multivariate analysis for LSR reduction of lumber spine and femoral neck BMD

Nagelkerke R^2^: 0.10	Lumber spine	
	Odds ratio	p value
Average dose of prednisolone	1.22 (1.02–1.50)	0.03
Type of anti-osteoporosis drugs	1.52 (0.87–2.65)	0.14
CRP	1.46 (0.95–2.25)	0.08

Nagelkerke R^2^: 0.08	Femoral neck	
	Odds ratio	p value
Sex	2.37 (0.82–6.90)	0.11
Disease duration	0.95 (0.91–0.10)	0.04
Average dose of prednisolone	1.13 (0.97–1.32)	0.11

The stepwise binomial logistic regression detected high dose of prednisolone use is a risk factors for 12-month LSR of lumbar spine (≤ − 2.4%). The stepwise binomial logistic regression analysis detected short disease duration is a risk factors for 12-month LSR of femoral neck (≤ − 1.9%)

*LSR* least significant reduction, *BMD* bone mineral density, *CRP* serum C-reactive protein concentration

### Discussion

Advanced age, female sex, longer disease duration, history of past thoracic or lumbar vertebral fractures, higher Steinbrocker classification, and lower BMI were previously determined to be associated with low femoral neck BMD in patients with RA treated with bDMARDs [18]. As these factors were correlated with a one-time examination of BMD, there is a possibility that other factors may confound those results. In this study, we examined LSR of BMD in the lumbar spine and femoral neck in RA patients treated with bDMARDs as measured at two time points. In this report, we showed that a high dose of methylprednisolone is a risk factor for LSR of BMD in the lumbar spine. This finding is consistent with previous studies [18, 26, 27]. Several studies showed that glucocorticoid therapy increase intensely risk of vertebral fracture [28–30]. Mori et al. reported in a multivariate linear regression analysis that longer RA disease duration was significantly related to the loss of BMD in the femoral neck and total femur [31]. Our findings showed that a risk factor for femoral neck BMD reduction was a short disease duration. There is a discrepancy between our results and the findings of previous reports [18, 31]. Bone loss was observed in patients with recently diagnosed rheumatoid arthritis, suggesting that the start of the femoral neck bone loss may be much earlier than previously anticipated [32].

It has been reported that bisphosphonates prevent bone loss of the lumbar spine and femoral neck in RA patients treated with bDMARDs [33, 34]. Although we could not detect the type of anti-osteoporosis drug as a significant risk factor, anti-osteoporosis drug therapy is necessary for some patients receiving bDMARDs. Further studies are needed to determine which anti-osteoporosis drugs are suitable to increase BMD in RA patients being treated with bDMARDs.

### Conclusions

Our findings showed that a risk factor for femoral neck BMD reduction was a short disease duration. These findings suggest that RA patients receiving treatment with bDMARDs may benefit from earlier osteoporosis treatments to prevent femoral neck bone loss.

#### Limitations

Our study has several limitations. Data collection was retrospective. The duration and kinds of bDMARDs used varied. It is better to examine BMD when patients begin taking bDMARDs. The Binary logistic regression analysis causes loss of information as it analyses a dichotomous outcome variable rather than the continuous bone loss measurement. We did not measure changes in biomarkers associated with bone remodeling in the blood or urine. Although prior studies did not find a difference in the levels of bone turnover markers after 1 year of treatment with bDMARDs [13, 35], we are now examining longitudinal changes in BMD and bone turnover markers in a prospective study.

## Additional file

**Additional file 1: Figure S1.** These scatter plot show the reduction of BMD in lumber spine (A) and femoral neck (B).

## Abbreviations

RA: rheumatoid arthritis
bDMARDs: biological disease-modifying anti-rheumatic drugs
LSR: least significant reduction
RANKL: receptor activator of nuclear factor-kappa B ligand
BMD: bone mineral density
DXA: dual X-ray absorptiometry
BMI: body mass index
CRP: serum C-reactive protein
DAS28-CRP: Disease Activity Score 28 CRP
SDAI: simplified disease activity index
TNFα: tumor necrosis factor-alpha
PTH: parathyroid hormone

## References

[1] Lodder MC, Haugeberg G, Lems WF, Uhlig T, Orstavik RE, Kostense PJ, Dijkmans BA, Kvien TK, Woolf AD (2003). Radiographic damage associated with low bone mineral density and vertebral deformities in rheumatoid arthritis: the Oslo-Truro-Amsterdam (OSTRA) collaborative study. Arthritis Rheum.

[2] Haugeberg G, Uhlig T, Falch JA, Halse JI, Kvien TK (2000). Bone mineral density and frequency of osteoporosis in female patients with rheumatoid arthritis: results from 394 patients in the Oslo County Rheumatoid Arthritis register. Arthritis Rheum.

[3] Dimitroulas T, Nikas SN, Trontzas P, Kitas GD (2013). Biologic therapies and systemic bone loss in rheumatoid arthritis. Autoimmun Rev.

[4] Emery P, Dorner T (2011). Optimising treatment in rheumatoid arthritis: a review of potential biological markers of response. Ann Rheum Dis.

[5] Venkateshan SP, Sidhu S, Malhotra S, Pandhi P (2009). Efficacy of biologicals in the treatment of rheumatoid arthritis. A meta-analysis. Pharmacology.

[6] Keystone E (2009). Recent concepts in the inhibition of radiographic progression with biologics. Curr Opin Rheumatol.

[7] Sakthiswary R, Das S (2013). The effects of TNF alpha antagonist therapy on bone metabolism in rheumatoid arthritis: a systematic review. Curr Drug Targets.

[8] Roussy JP, Bessette L, Bernatsky S, Rahme E, Lachaine J (2013). Biologic disease-modifying anti-rheumatic drugs and the risk of non-vertebral osteoporotic fractures in patients with rheumatoid arthritis aged 50 years and over. Osteoporos Int.

[9] Kawai VK, Grijalva CG, Arbogast PG, Curtis JR, Solomon DH, Delzell E, Chen L, Ouellet-Hellstrom R, Herrinton L, Liu L (2013). Initiation of tumor necrosis factor alpha antagonists and risk of fractures in patients with selected rheumatic and autoimmune diseases. Arthritis Care Res.

[10] Confavreux CB, Chapurlat RD (2011). Systemic bone effects of biologic therapies in rheumatoid arthritis and ankylosing spondylitis. Osteoporos Int.

[11] Hoff M, Kvien TK, Kalvesten J, Elden A, Haugeberg G (2009). Adalimumab therapy reduces hand bone loss in early rheumatoid arthritis: explorative analyses from the PREMIER study. Ann Rheum Dis.

[12] Haugeberg G, Conaghan PG, Quinn M, Emery P (2009). Bone loss in patients with active early rheumatoid arthritis: infliximab and methotrexate compared with methotrexate treatment alone. Explorative analysis from a 12-month randomised, double-blind, placebo-controlled study. Ann Rheum Dis.

[13] Marotte H, Miossec P (2008). Prevention of bone mineral density loss in patients with rheumatoid arthritis treated with anti-TNFalpha therapy. Biologics.

[14] Ammann P, Rizzoli R, Bonjour JP, Bourrin S, Meyer JM, Vassalli P, Garcia I (1997). Transgenic mice expressing soluble tumor necrosis factor-receptor are protected against bone loss caused by estrogen deficiency. J Clin Investig.

[15] Neogi T, Aletaha D, Silman AJ, Naden RL, Felson DT, Aggarwal R, Bingham CO, Birnbaum NS, Burmester GR, Bykerk VP (2010). The 2010 American College of Rheumatology/European League Against Rheumatism classification criteria for rheumatoid arthritis: phase 2 methodological report. Arthritis Rheum.

[16] Fransen J, Creemers MC, Van Riel PL (2004). Remission in rheumatoid arthritis: agreement of the disease activity score (DAS28) with the ARA preliminary remission criteria. Rheumatology.

[17] Felson DT, Smolen JS, Wells G, Zhang B, van Tuyl LH, Funovits J, Aletaha D, Allaart CF, Bathon J, Bombardieri S (2011). American College of Rheumatology/European League against Rheumatism provisional definition of remission in rheumatoid arthritis for clinical trials. Ann Rheum Dis.

[18] Takahashi K, Setoguchi T, Tawaratsumida H, Arishima Y, Tominaga H, Ishidou Y, Nagano S, Shigemizu S, Aoki N, Akimoto M (2015). Risk of low bone mineral density in patients with rheumatoid arthritis treated with biologics. BMC Musculoskelet Disord.

[19] Nawata H, Soen S, Takayanagi R, Tanaka I, Takaoka K, Fukunaga M, Matsumoto T, Suzuki Y, Tanaka H, Fujiwara S (2005). Guidelines on the management and treatment of glucocorticoid-induced osteoporosis of the Japanese Society for Bone and Mineral Research (2004). J Bone Miner Metab.

[20] Suzuki Y, Nawata H, Soen S, Fujiwara S, Nakayama H, Tanaka I, Ozono K, Sagawa A, Takayanagi R, Tanaka H (2014). Guidelines on the management and treatment of glucocorticoid-induced osteoporosis of the Japanese Society for Bone and Mineral Research: 2014 update. J Bone Miner Metab.

[21] Orimo H, Hayashi Y, Fukunaga M, Sone T, Fujiwara S, Shiraki M, Kushida K, Miyamoto S, Soen S, Nishimura J (2001). Diagnostic criteria for primary osteoporosis: year 2000 revision. J Bone Miner Metab.

[22] Soen S, Fukunaga M, Sugimoto T, Sone T, Fujiwara S, Endo N, Gorai I, Shiraki M, Hagino H, Hosoi T (2013). Diagnostic criteria for primary osteoporosis: year 2012 revision. J Bone Miner Metab.

[23] Diez-Perez A, Adachi JD, Agnusdei D, Bilezikian JP, Compston JE, Cummings SR, Eastell R, Eriksen EF, Gonzalez-Macias J, Liberman UA (2012). Treatment failure in osteoporosis. Osteoporos Int.

[24] Shiraki M, Ueda S, Sugimoto T, Kuroda T, Nakamura T (2016). Treatment responses with once-weekly teriparatide therapy for osteoporosis. Osteoporos Int.

[25] Fischer KE, Rogowski WH, Leidl R, Stollenwerk B (2013). Transparency vs. closed-door policy: do process characteristics have an impact on the outcomes of coverage decisions? A statistical analysis. Health Policy.

[26] Shibuya K, Hagino H, Morio Y, Teshima R (2002). Cross-sectional and longitudinal study of osteoporosis in patients with rheumatoid arthritis. Clin Rheumatol.

[27] Cortet B, Flipo RM, Blanckaert F, Duquesnoy B, Marchandise X, Delcambre B (1997). Evaluation of bone mineral density in patients with rheumatoid arthritis. Influence of disease activity and glucocorticoid therapy. Rev Rhum Engl Ed.

[28] De Vries F, Bracke M, Leufkens HG, Lammers JW, Cooper C, Van Staa TP (2007). Fracture risk with intermittent high-dose oral glucocorticoid therapy. Arthritis Rheum.

[29] van Staa TP, Leufkens HG, Cooper C (2002). The epidemiology of corticosteroid-induced osteoporosis: a meta-analysis. Osteoporos Int.

[30] van Staa TP (2006). The pathogenesis, epidemiology and management of glucocorticoid-induced osteoporosis. Calcif Tissue Int.

[31] Mori Y, Kuwahara Y, Chiba S, Kogre A, Baba K, Kamimura M, Itoi E (2017). Bone mineral density of postmenopausal women with rheumatoid arthritis depends on disease duration regardless of treatment. J Bone Miner Metab..

[32] Kleyer A, Schett G (2014). Arthritis and bone loss: a hen and egg story. Curr Opin Rheumatol.

[33] Jensen TW, Hansen MS, Horslev-Petersen K, Hyldstrup L, Abrahamsen B, Langdahl B, Zerahn B, Podenphant J, Stengaard-Petersen K, Junker P (2014). Periarticular and generalised bone loss in patients with early rheumatoid arthritis: influence of alendronate and intra-articular glucocorticoid treatment. Post hoc analyses from the CIMESTRA trial. Ann Rheum Dis.

[34] Krieckaert CL, Nurmohamed MT, Wolbink G, Lems WF (2013). Changes in bone mineral density during long-term treatment with adalimumab in patients with rheumatoid arthritis: a cohort study. Rheumatology.

[35] Marotte H, Pallot-Prades B, Grange L, Gaudin P, Alexandre C, Miossec P (2007). A 1-year case–control study in patients with rheumatoid arthritis indicates prevention of loss of bone mineral density in both responders and nonresponders to infliximab. Arthritis Res Ther.

