# Supplementary figures and images for: Risk factors for bone loss in patients with rheumatoid arthritis treated with biologic disease-modifying anti-rheumatic drugs
Source: BMC Res Notes. 2017 Dec 21;10:765. doi: 10.1186/s13104-017-3086-7 (PMC5740597; doi:10.1186/s13104-017-3086-7)

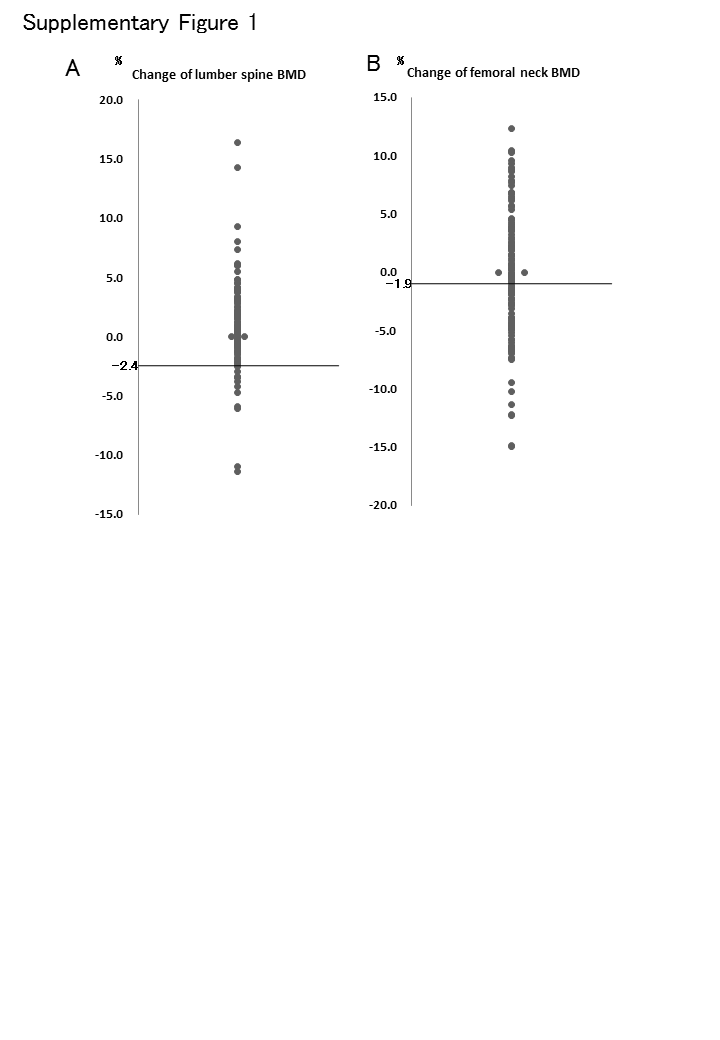

Supplement: Supplementary file 1 — Additional file 1: Figure S1. These scatter plot show the reduction of BMD in lumber spine (A) and femoral neck (B). [file 13104_2017_3086_MOESM1_ESM.tif]
